# Supplementary material for: Coronavirus membrane protein with a fluorescent protein tag enables particle tracking for the study of virus assembly and egress in live cells
Source: J Virol. 2026 Jul 6;100(8):e02228-25. doi: 10.1128/jvi.02228-25 (PMC13483320; doi:10.1128/jvi.02228-25)
Supplement: Supplementary file 1 [file jvi.02228-25-s0001.docx]

**Supplemental**

**Fig. S1**. M-GFP protein remains intact when expressed. (A) L2 cells were transfected with untagged WT M or GFP; M-GFP with a V5 tag at the amino end; GFP or M-GFP with a V5 tag at the carboxy end. At 24 h after transfection cell lysates were immunoblotted and probed with antibodies against GFP or V5. When probed with anti-GFP and anti-V5, the 5’ V5 M-GFP and the 3’ V5 M-GFP show bands 37kDa and 50 kDa (lanes 5 and 7). (B) L2 cells were transfected with M-GFP with the amino V5 tag (top row) or amino V5 tag (bottom row). At 24 hours after transfection, cells were immunolabeled for WT M and V5, imaged by confocal. Typical fields are shown selected from at least 3 fields acquired from each of 2 independent experiments. White=nuclei (DAPI); green=M-GFP; red=WT M; blue=V5. Scale bar = 10 µm.
