# Supplementary material for: Coronavirus membrane protein with a fluorescent protein tag enables particle tracking for the study of virus assembly and egress in live cells
Source: J Virol. 2026 Jul 6;100(8):e02228-25. doi: 10.1128/jvi.02228-25 (PMC13483320; doi:10.1128/jvi.02228-25)
Supplement: Supplementary file 2 [file jvi.02228-25-s0003.docx]

**Supplemental Movie Legends**

**Supplemental movie 1**

M-GFP used to track the progression of MHV infection in live cells. L2 cells were infected with M-GFP MHV. Nuclei were labeled with Hoechst. Cells were imaged by confocal. Z-stacks were collected every 2 minutes from 5-10 hpi. Movie is a 3D rendered view from the perspective of looking down on the cells from above. Green=GFP; blue=nuclei (Hoechst).

**Supplemental movie 2**

M-GFP allows imaging of assembled MHV virus trafficking at and near the plasma membrane. 17Cl1 cells were infected with M-GFP MHV and imaged live by TIRF microscopy at 6-7hpi. TIRF was collected in the GFP channel and overlaid on differential interference contrast, still image. Timelapse images were collected for up to 2.5 minutes per field. A typical timelapse is shown, selected from at least 5 datasets taken from each of 3 independent experiments.

**Supplemental movie 3**

Tracking of virus at and near the plasma membrane in 17Cl1 infected cells. Timelapse images collected by TIRF (as described in move 2 legend) were analyzed by Nikon Elements particle tracking module. Movie shows tracks detected from 60 seconds of data collection.
